# Aerosol-assisted CVD method for the synthesis of solid particles of t-YSZ-Fe_3_O_4_

**DOI:** 10.1016/j.mex.2022.101973

**Published:** 2022-12-16

**Authors:** K.I. Contreras-Vargas, A. Heiras-Trevizo, P. Amézaga-Madrid

**Affiliations:** Departamento de Física de Materiales, Centro de Investigación en Materiales Avanzados, S.C., Chihuahua, Chih 31136, México

**Keywords:** AACVD, Aerosol assisted-CVD, t-YSZ-Fe_3_O_4_ particles, Solid particles, Aerosol-assisted CVD method for the synthesis of solid particles of t-YSZ-Fe_3_O_4_

## Abstract

This work details the production of solid composite particles by the aerosol-assisted chemical vapor deposition method. With this method it is feasible to produce at low temperature (450 °C) the tetragonal phase of zirconium oxide stabilizing it with yttrium oxide (YSZ) and cubic iron oxide (Fe_3_O_4_) at the same time. The particles have a solid morphology in which both metal oxides coexist without mixing. The average size of the obtained particles is 329 ± 81 nm, moreover, each particle is formed by thousands of crystallites of size 2 ± 0.5 nm. The formation of solid structures is due to the amount of Zr and Y found in each particle. These particles can be applied as reinforcements of metallic structures.

• A simple and low-cost method for producing composite particles to be applied as reinforcing agents for metal structures.
• The particles are formed by two phases of tetragonal yttria-stabilized zirconia (t-YSZ) and cubic Fe_3_O_4_, which was synthesized following a one-step process via the aerosol-assisted chemical vapor deposition method (AACVD).
• The tetragonal phase of ZrO_2_ is obtained at 450 °C stabilizing it with ∼3.8% of yttrium oxide.

## Graphical abstract

**Figure:**
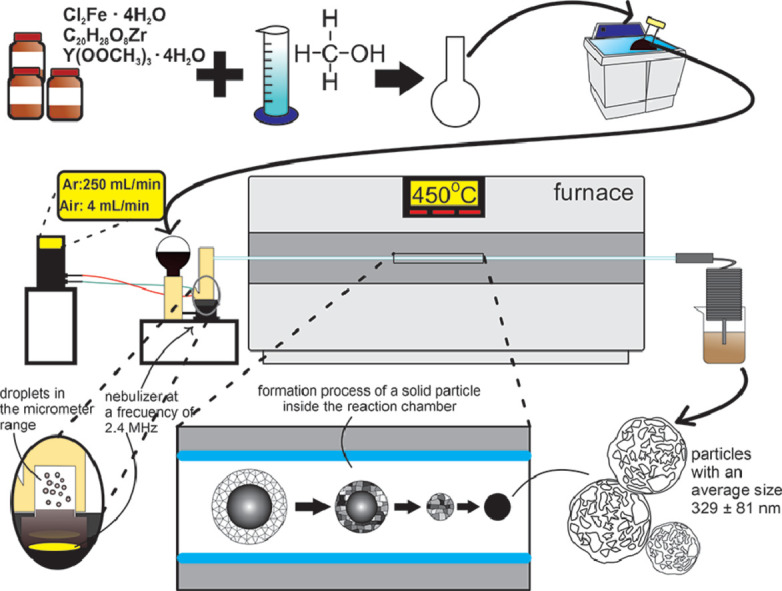

Specifications table

**Table:** 

Subject Area:	Materials Science
More specific subject area:	Synthesis and characterization of nanostructured materials, particularly metal oxides.
Protocol name:	Aerosol-assisted CVD method for the synthesis of solid particles of t-YSZ-Fe_3_O_4_
Reagents/tools:	
Experimental design:	The synthesis of nanostructured solid particles of zirconium oxide stabilized with yttrium oxide and magnetite by the AACVD technique is described in detail; the system and all its accessories are shown part by part. The obtaining of these particles by a simple methodology and relatively easy to install in a laboratory is explained step by step.
Trial registration:	Not applicable
Ethics:	Not applicable

Value of the protocol

• AACVD method is a low-cost, easily manipulated, simple and scalable, without the need for complex infrastructure.
• The composite material is produced in a single step, continuously without requiring a vacuum system, process control agents, additives, templates, or pH controllers.
• The tetragonal phase of zirconium oxide is obtained at a low temperature, around 450 °C.

Description of protocol

## Details and fundamentals of the AACVD method

Aerosol-assisted chemical vapor deposition (AACVD) is one of the physicochemical methods that allows for obtaining nanostructured materials with reproducibility and controlled characteristics [1], [2], [3], [4], [5], [6], [7], [8], [9], [10], [11], [12], [13], [14], [15], [16], [17] which is a variant of the conventional CVD process. This method is mostly used for the synthesis of thin films and powders and also because the formation of nanostructures takes place at or near atmospheric pressure, and usually at ambient air. With this technique it is possible to obtain nanostructures with different geometries such as thin films [6,11], nanorods [8,9], nanowires [9,16], nano-alloy films [15], nanoparticles [1,^2^,17] for different and diverse applications in photocatalysis [12,13], production of hydrogen [15,16], adsorption processes and water treatment [1,^2^,^14^,17], as reinforcements of metallic structures [4,5], in solar cells as selective absorbing materials [10] and transparent conductive materials [7].The materials synthesized by AACVD are produced in a single step, in a continuous and relatively simple way, unlike other techniques that fabricate similar nanostructures in several stages, require a larger amount of reagents, pH control agents and longer synthesis time [18], [19], [20]. Thus, automatically reducing the cost in terms of infrastructure investment, reduction of energy costs, extra reagents, time, etc.

In this case, precursor solutions composed of organometallic or inorganic salts dissolved in organic or aqueous solvents are used to synthesize particles. The technique consists of producing a cloud of the precursor solution, which is transported by a vector gas or gas mixture to the interior of a tubular reactor, located inside a temperature-controlled chamber (furnace). Due to the temperature of the reactor, the decomposition of the precursor's salts and the formation of the desired material takes place. Directly connected to the tubular reactor at the exit of the chamber a collection device is used to collect the powder, which consists of a coil that helps to diminish the velocity of the formed particles and also cools the carrier gas flow with the material to be collected in a beaker with a recollection liquid. The temperature of the furnace is the fundamental parameter that provides the thermal energy necessary to control the synthesis of the material. This temperature depends on the material to be obtained and the precursors to be used.

The synthesis process begins when the cloud of droplets enters the tubular reactor and is heated in the reaction chamber. The following steps must occur successively: (a) evaporation of the solvent; (b) nucleation of precursor salts; (c) melting, or eventual sublimation of the precursor compound; (d) evaporation or thermal decomposition, (e) chemical reaction of the elements of interest and the formation of the required material. This methodology allows obtaining powders in the form of particles with hollow or solid morphology. The composition of the precursor solution plays an important factor in the morphology of the resulting material. The physical basis for the generation of hollow or solid particles is reported in detail in [5,^17^,[21], [22], [23].

## Description of the experimental AACVD system

**Fig. 1:**
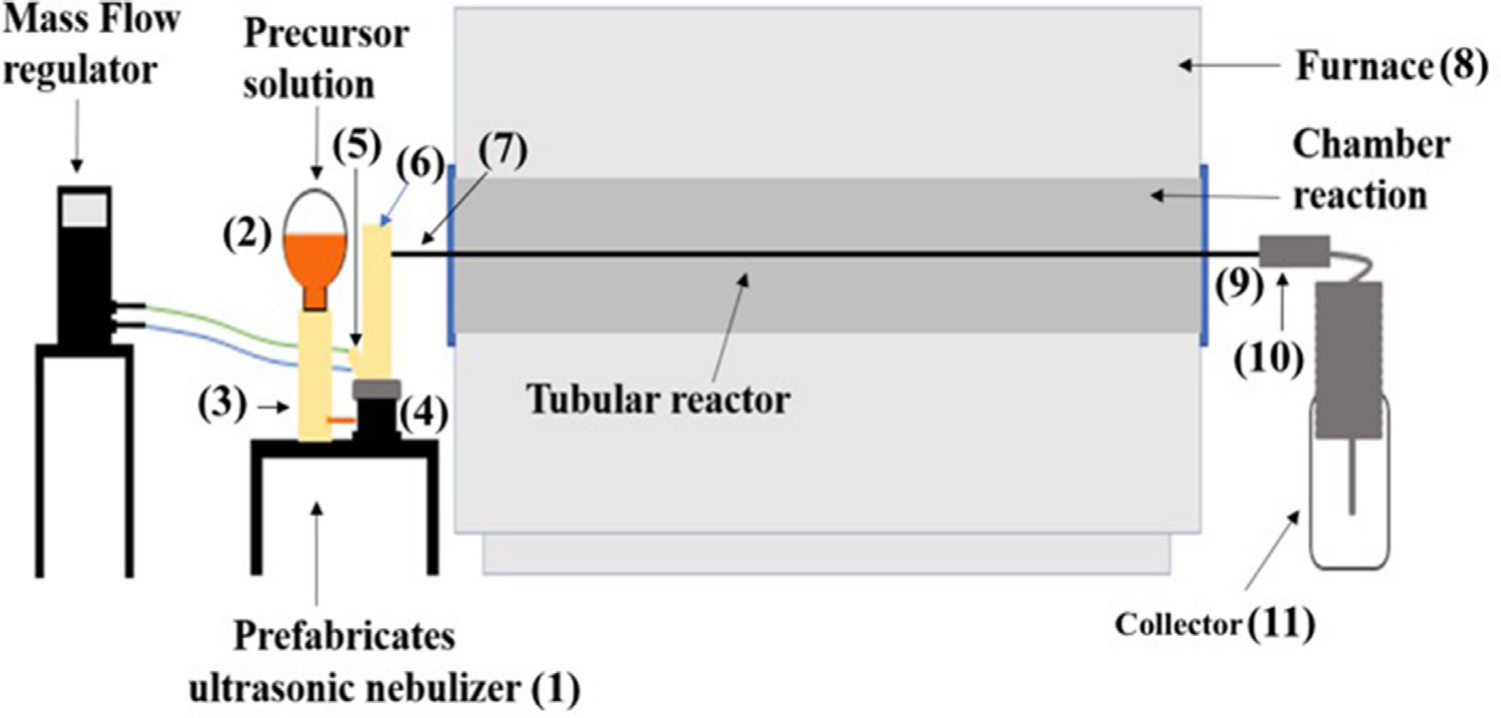
Schematic diagram of the AACVD system, showing the principal components required to synthesize the particles.

In this section, a detailed description of an AACVD system in the synthesis of the particles with the details of the components as well as how they are interconnected. Fig. 1 shows a schematic diagram of the AACVD system used to produce the t-YSZ-Fe_3_O_4_ particles. The principal components of the system are: (a) the nebulizer (1), (b) a tubular furnace (8), and (c) the particle collection device (11).

(a) The nebulizer is of the ultrasonic type and operates at a frequency of 2.4 MHz. The principal advantage of this type of nebulizer is that it provides a narrow size distribution of the precursor solution droplets (diameter around 2 μm) from which the particles will be synthesized. With a narrow size distribution of the droplets, an optimal deposition condition (such as deposition temperature, carrier gas type and flow, and precursor solution composition) can be attained for these droplets of a particular diameter. Otherwise, with a wide size distribution of the droplets, the optimal deposition conditions are different for droplets of very different sizes. In this case, the synthesis will develop under non-optimal conditions for many sizes of the droplets, only droplets of around some particular size will have favorable conditions to produce the desired material with the required microstructure and properties. The nebulizer consists of a precursor solution container (3) and its level regulator (2), a nebulization chamber (4), an inlet of the carrier gas (5) which is controlled, by a mass flow regulator (6), and finally a coupling (7) with the borosilicate tube, which is inserted inside the tubular furnace. The core of this device is inside the nebulization chamber (4), which is filled with the precursor solution, and in contact with a piezoelectric crystal, located at the bottom of the chamber. The piezoelectric vibrate at a high frequency (∼ MHz) generating ultrasonic waves within the volume of the precursor solution. The geometry of the chamber causes a concentration of the ultrasonic waves at a certain distance from the piezoelectric surface, the optimum generation of droplets occurs if the surface of the solution coincides with the distance where the concentration of the waves takes place. Under these circumstances, thousands of micrometric droplets will be generated at the surface of the solution; the size of the droplets depends on the frequency of the vibration, density and surface tension of the solution. Finally, the cloud of droplets is conveyed by the carrier gas (5) and injected into the borosilicate tube (7).
(b) The tubular furnace (8). The borosilicate tube or tubular reactor (7) is introduced inside of the tubular furnace (8). The tubular reactor must exit from the front and rear of the furnace to allow coupling with the nebulizer (7) and the collection system (9), respectively.
(c) The particle collection device (11) consists of a stainless-steel tubular coil (10), connected on one side to the outlet of the tubular reactor (9) and the other end is immersed into some appropriate liquid inside the beaker (11). In the case of the t-YSZ-Fe_3_O_4_, the beaker contains tri-distilled water. Another important function of the liquid inside of the collector flask is that it does not allow the oxygen from the environment to pass into the tubular reactor; it only allows the exit of the system's product gasses, constituting what is called a liquid trap.

Once the particles are obtained, they are washed with tri-distilled water and centrifuged to obtain the material using an ultrasonic sonicator (Cole Parmer), a Clinical 200 centrifuge (VWR) and a microcentrifuge (Thermoscientific) respectively. Subsequently, the powder is dried and stored in a desiccator for future application.

**Fig. 2:**
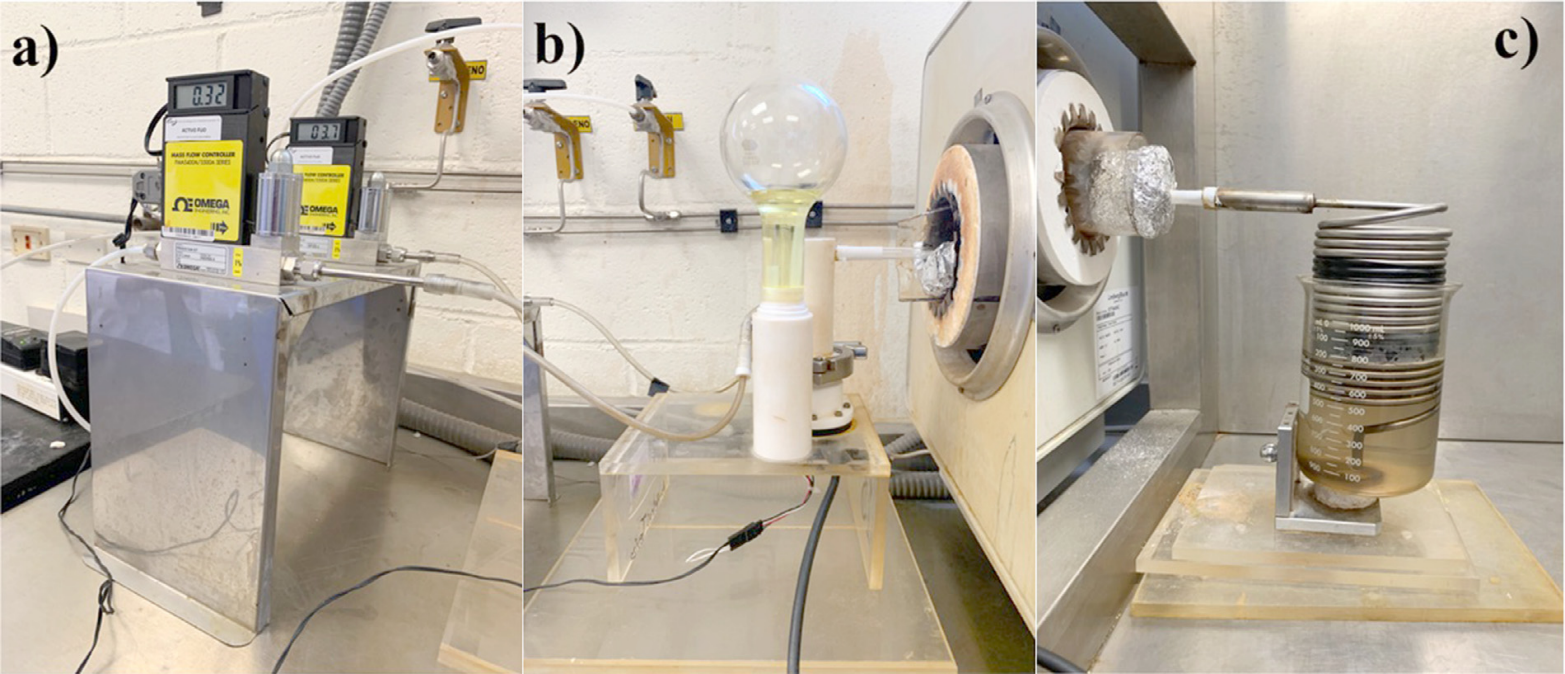
The fundamental components of the actual AACVD system. (a) The mass flow regulators. (b) The self-fabricated ultrasonic nebulizer and the precursor solution container are already assembled with the tubular reactor inserted inside the furnace. (c) The furnace outlet shows the tubular reactor coupled with the collecting system.

Fig. 2 shows the components of the actual AACVD system, the mass flow controllers (Fig. 2a) which are connected to the nebulizer (Fig. 2b), and the collector system (Fig. 2c). It is worth mentioning that this laboratory-level AACVD system does not require a complex infrastructure to be implemented (such as a vacuum system, process control agents, or plasma systems), making it a low-cost, easily controlled, relatively simple, method for producing high quality oxides and noble metals, and additionally scalable at an industrial level.

## Materials and equipment necessary to synthesize the particles of t-YSZ-Fe_3_O_4_

Table 1 shows the reagents needed for the synthesis of t-YSZ-Fe_3_O_4_

**Table 1:** Reagents used for the synthesis of the t-YSZ-Fe_3_O_4_.

Reagent	Chemical formula	Molecular weight	Supplier	CAS
Tridistilled water	H_2_O	18	J.T. Baker	7732-18-5
Air gas	–	–	–	–
Argon gas, grade 4.8	Ar	39.95	Praxair	–
Methanol	CH_3_ · OH	32	J.T. Baker	67-56-1
Iron chloride tetrahydrate	Cl_2_Fe · 4H_2_O	198.81	Sigma Aldrich	13478-10-9
Zirconium acetylacetonate	C_20_H_28_O_8_Zr	487.66	Sigma Aldrich	17501-44-9
Yttrium acetate	Y(OOCH_3_)_3_ · 4H_2_O	338.1	Alfa Aesar	23363-14-6

## Experimental procedure

1. The synthesis begins with the preparation of the precursor solution containing the three salts of the elements of interest: zirconium (Zr), Y (yttrium), and (Fe) iron (see Table 1). The salts are dissolved in methanol. The final molar concentration of the precursor solution is 0.1 mol.dm^−3^ with a stoichiometric ratio of Fe:Zr:Y of 60:39:1 respectively. It is very important to obtain the total dissolution of the three precursor salts used since it is a determining factor for obtaining homogeneous materials both in composition and morphology.
2. Then the three parts of the AACVD system are coupled together: nebulizer-furnace/tubular reactor-collector system, it is important that the couplings are completely airtight, to avoid the intake of atmospheric air or possible leakage of the droplet cloud that influences the composition of the synthesized material.
3. Following the formulation of the precursor solution and assembling of the system, the precursor solution is placed inside the solution container and nebulizer chamber.
4. Subsequently, the introduction of the carrier gas mixture is started. The carrier gas is composed of a mixture of an inert gas (argon) and an oxidizing gas (filtered clean and dry air). The flows of the inert and oxidizing gas must be controlled with precision by employing mass flow regulators. The flow rates are 250 mL/min for the argon gas and 4 mL/min for the oxidizing gas (air). The composition and flow rate of the aerosol carrier gas is an important parameter since it allows to control of the nature and microstructural characteristics of the material to be obtained, this means generating the metal oxide phases of interest, in this case, t-YSZ-Fe_3_O_4_.
5. Subsequently, the tubular furnace temperature is set at 450 °C.
6. Once the synthesis conditions mentioned above are reached, the nebulization of the precursor solution is started in the nebulizer, simultaneously the aerosol cloud is dragged by the carrier gas and introduced into the tubular reactor, being heated inside the tubular furnace. With heating, important stages occur that will determine the morphology and final structure of the materials, one of them is the evaporation of the drop where physical processes are involved, such as the diffusion rate of the solute and the evaporation rate of the solvent, which will allow the physical and chemical transformations that generate the t-YSZ-Fe_3_O_4_ particles, phenomena already detailed in [5,^17^,[21], [22], [23].

**Fig. 3:**
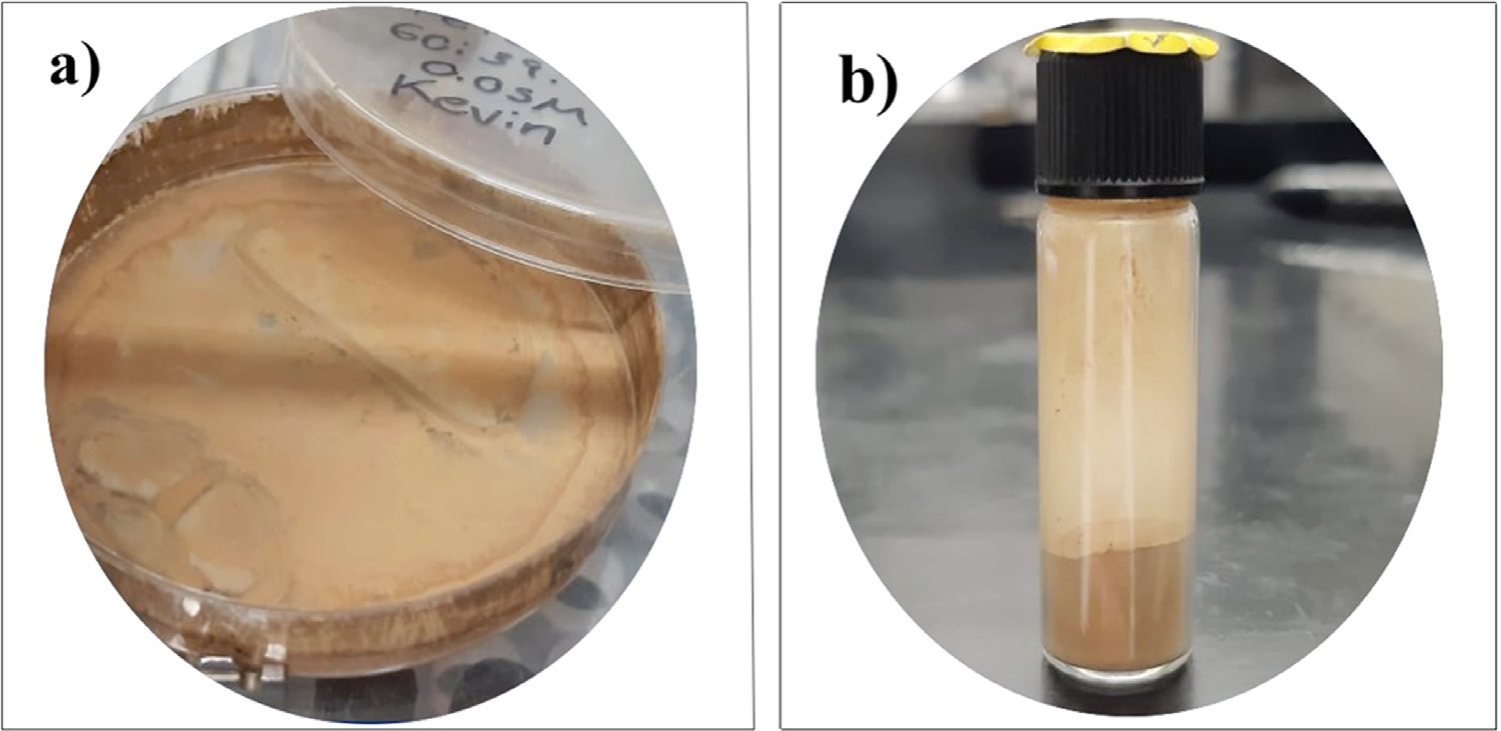
Powders (t-YSZ-Fe_3_O_4_ particles) recollected after synthesis. (a) The particles are collected in a Petri dish for subsequent drying. (b) Then particles are stored in a glass vial with a screw cap for further use.

The particles generated continue to be transported by the flow of the carrier gas towards the outside of the furnace and introduced into the collection liquid of the recovery system. Once the nebulization has started, the synthesis time is continued until the desired amount of the t-YSZ-Fe_3_O_4_ particles is obtained. As supplementary material, video 1 is presented, which shows the AACVD system in real operation. At the end of the process, the equipment is turned off, the collection container is removed, and the particles obtained by centrifugation are recovered, collected in a Petri dish, placed in a desiccator for drying, and then deposited in a vial with a screw cap for storage. In addition, Fig. 3 shows the powder obtained with a brown coloration deposited in a petri dish after centrifugation and drying and then placed in a glass vial with a screw cap for storage.

**Fig. 4:**
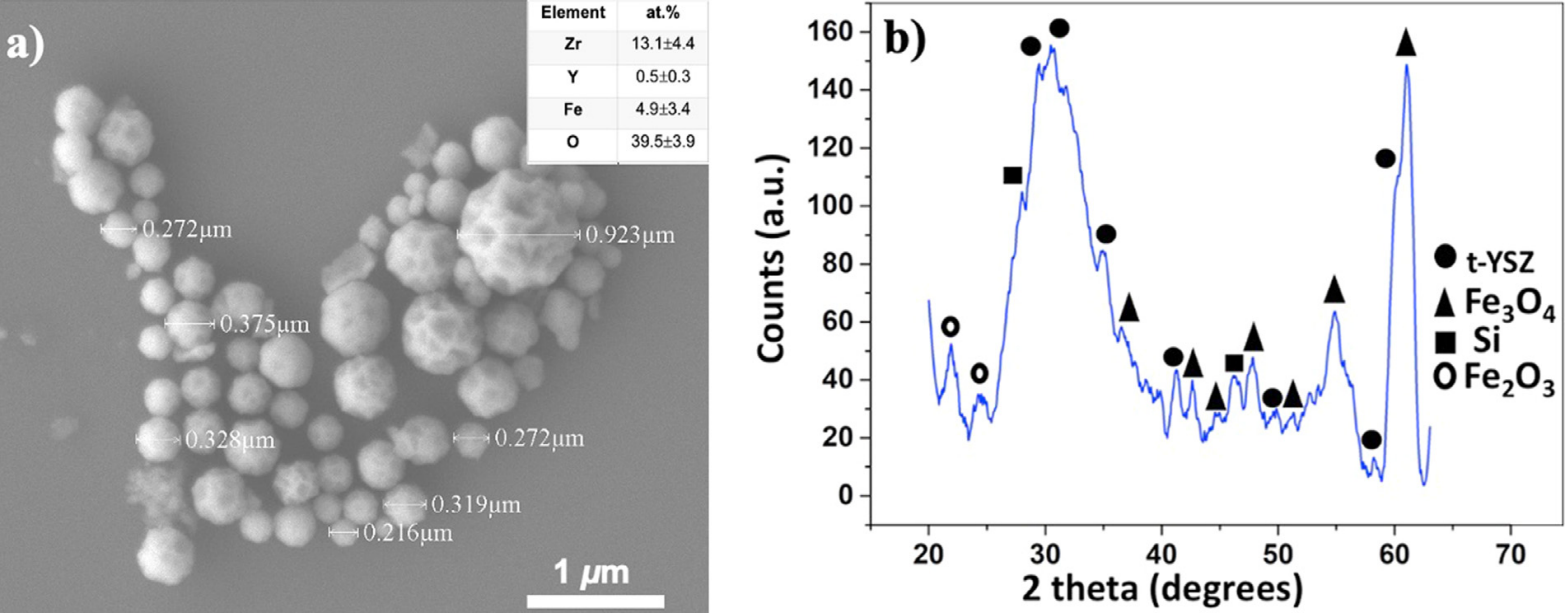
(a) SEM micrograph showing the solid morphology of the particles, the elemental composition is inserted in the figure [5]. (b) Shows the diffraction pattern and the crystalline phases present in the material obtained [5].

Finally, Fig. 4a, shows a micrograph obtained by SEM, which presents the morphology of the particles, and inserted in Fig. 4a, there is a table showing the elemental composition in atomic percentage, the presence of the elements of interest Zr, Y, Fe and O is observed. The Fig. 4b, shows the diffraction pattern indicating the crystalline phases obtained, YSZ in tetragonal phase (JCPDF 00–083–0113; 01–081–1317) [24], Fe_3_O_4_ (JCPDF 019–0629) [24], traces of Fe_2_O_3_ (JCPDF 01–088–2359) [24] and Si, the substrate where the sample was prepared for analysis) [5].

## Method validation

A powdered composite material comprised of mainly t-YSZ and cubic Fe_3_O_4_ was obtained using the reproducible AACVD synthesis method. Noteworthy, the material was synthesized in one step, at relatively low temperature (450 °C), and without the utilization of templates or the addition of pH control reagents, additives, and subsequent high-temperature annealing steps. The chemical reaction and the formation of the solid composite particles occur optimally at the established temperature. The performance of the AACVD equipment is very stable, as long as necessary maintenance services are provided. The efficiency of the system in a continuous operation is around 11 ± 1 mg h^−1^ of particles. The particles exhibited a solid-like structure with an irregular morphology at the surface. The experience in working with this AACVD system and method has been more than a decade. The validation of the method occurs when obtaining the nanostructured powder, with the characteristics and chemical phases proposed at the beginning.

## Declaration of Competing Interest

The authors declare that they have no known competing financial interests or personal relationships that could have appeared to influence the work reported in this paper.

## Data Availability

No data was used for the research described in the article.

## Appendix Supplementary materials

Image, video 1
